# Intriguing choroidal lesions in Birdshot chorioretinopathy: a diagnostic and clinical dilemma

**DOI:** 10.1186/s12348-025-00557-8

**Published:** 2025-12-22

**Authors:** Yann Bertolani, Tetiana Goncharova, Eric Kirkegaard-Biosca, Laura Distefano

**Affiliations:** Department of Ophthalmology, Vall d’Hebron University Hospital, Passeig de la Vall d’Hebron 129, Barcelona, 08035 Spain

**Keywords:** Uveal lymphoid hyperplasia, Ocular oncology, Intraocular mass, Birdshot chorioretinopathy, Choroiditis

## Abstract

**Purpose:**

To report a case of intriguing choroidal lesions in a patient with Birdshot chorioretinopathy.

**Case presentation:**

A case report of a 51-year-old female with previous medical history of Birdshot chorioretinopathy presenting with de novo choroidal lesions, diffuse choroidal thickening and serous retinal detachment in the right eye is presented. Several medical evaluation visits were conducted, including ophthalmological and hematological follow-up. The multimodal imaging was suggestive of a lymphoproliferative process, presumably uveal lymphoid hyperplasia and the patient was referred to Hematology. A systemic work-up was conducted to rule out systemic malignancy and the choroidal biopsy was declined by the patient. The patient was treated with a short course of steroids due to an unrelated Bell’s palsy, with complete resolution of the choroidal thickening and the neurosensory detachment. After a 4-year follow-up, the patient remained asymptomatic with no signs of relapse in the multimodal imaging.

**Conclusion:**

As underline by this case report, uveal lymphoid hyperplasia may be considered in patients with Birdshot chorioretinopathy, emphasizing the importance of individualized management and long-term follow-up in such complex clinical scenarios.

## Introduction

Birdshot chorioretinopathy (BC) is a bilateral posterior uveitis characterized by peripapillary and midperipheral choroidal lesions [1]. Although the etiology is unknown, an autoimmune response to the retinal S antigen is postulated, with HLA-A29 being a powerful associated genetic factor [1]. The subacute and insidious course of the disease may pose a significant diagnostic challenge as patients may complain of non-specific symptoms such as floaters, nyctalopia and visual loss [1, 2]. Long-term complications from BC include chorioretinal atrophy, chronic cystoid macular edema, cataract and glaucoma [1, 2].

Uveal lymphoid hyperplasia (ULH) is a rare and challenging clinical choroidal entity, composed of a polyclonal proliferation of B lymphocytes [3]. Formerly misnamed as benign reactive lymphoid hyperplasia, many cases showed low-grade lymphomatous transformation as suggested by histopathological and immunochemical analysis [4]. Being such an infrequent clinical entity, the diagnosis of ULH represents a complex clinical scenario with a broad differential diagnosis, including neoplastic, infectious and uveitic conditions such as BC [5]. Multimodal imaging including eye fundus, B-mode ultrasound, optical coherence tomography (OCT), fluorescein angiography (FA) and indocyanine green (ICG) is crucial in the differential diagnosis and therapeutic monitoring of choroidal diseases such as ULH and BC [6].

We present an exceptional case of BC that during follow-up developed unilateral intriguing choroidal lesions compatible with HLU, highlighting the diagnostic process, treatment approach and the clinical implications of this exceptional association.

## Case presentation

In 2004, a 34-year-old woman presented to the emergency room complaining of subacute visual loss in both eyes (OU). Her personal history included antiphospholipid syndrome under anticoagulant treatment. Upon the initial evaluation, her best-corrected visual acuity (BCVA) was 20/30 (Snellen chart) in the right eye (OD) and 20/50 in the left eye (OS). The fundus exam depicted bilateral optic disc edema and a peripapillary splinter hemorrhage in the OS. Given these findings, the patient was referred to Neurology and a lumbar puncture was performed, with a normal CSF study. Five days later, the patient was re-evaluated by Ophthalmology due to worsening of BVCA in the OS to 20/200.

New peripapillary choroidal lesions were noted in OU, predominantly in the OS. A study was conducted to rule out infectious and inflammatory etiologies, which revealed a positive HLA-A29 marker. Additionally, FA evidenced late hyperfluorescent peripapillary lesions in OU, predominantly in OS and venous leakage in OU. All in all, the findings and the clinical scenario were suggestive of BC. Treatment was started with 1 mg/kg/day prednisone with progressive tapering for six months, with correct resolution of the symptoms and an almost complete disappearance of the choroidal lesions. No other immunosuppressive agent was used due to the sustained clinical response and the absence of relapse. At the end of treatment, BVCA was 20/25 in OU, with residual choroidal scars in the OS. Eventually, the patient underwent annual follow-up, with stability of the ophthalmological examination.

**Fig. 1:**
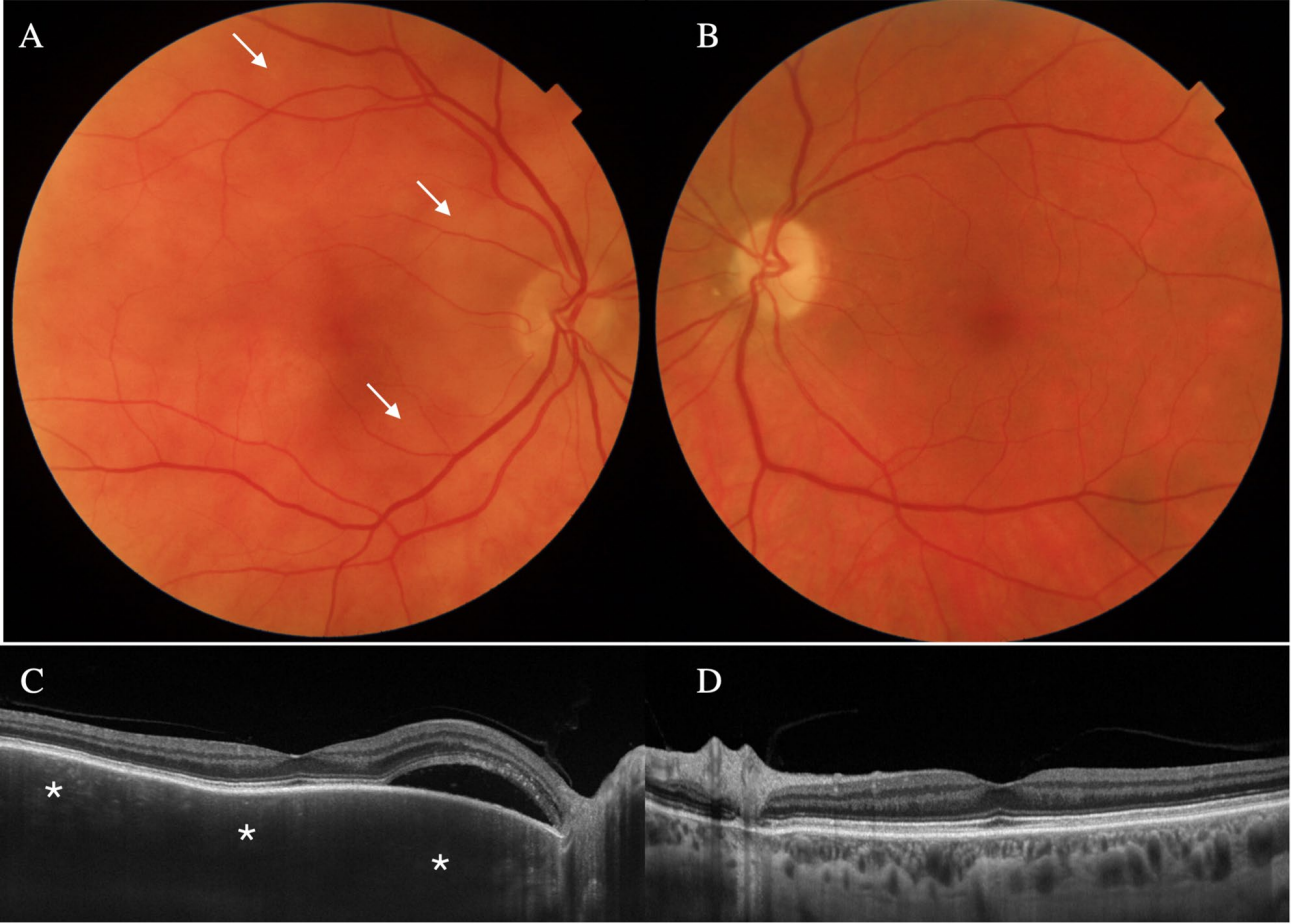
Presumed uveal lymphoid hyperplasia at presentation in the right eye (**A**) Retinography of the right eye with creamy multifocal choroidal lesions (arrows) (**B**) Retinography of the left eye with no abnormal findings (**C**) Macular optical coherence tomography of the right eye at presentation with choroidal thickening (asterisks), compression of the choriocapillaris and peripapillary serous retinal detachment (**D**) Macular optical coherence tomography of the left eye with no abnormalities

**Fig. 2:**
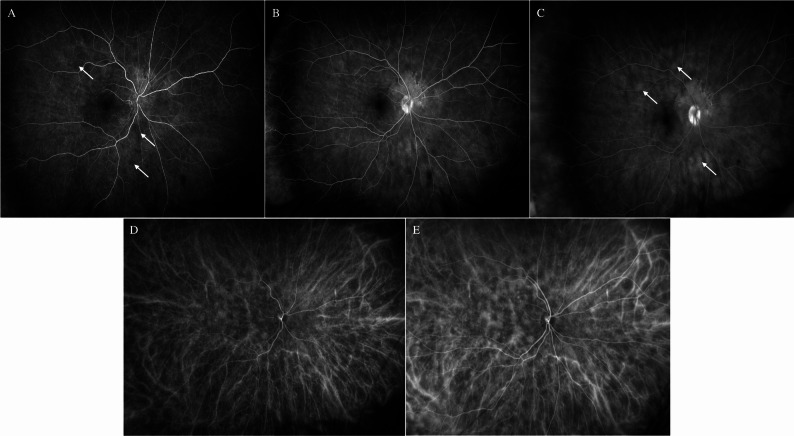
Multimodal imaging of presumed uveal lymphoid hyperplasia in the right eye (**A**) Early arteriovenous phase of the fluorescein angiography with leakage at the peripapillary level and hypofluorescence secondary to choroidal lesions (arrows) (**B**) Venous phase of the fluorescein angiography with increased peripapillary and hypofluorescence secondary to choroidal lesions (**C**) Late phase of the fluorescein angiography with peripapillary and capillary leakage (arrows) and hypofluorescence secondary to choroidal lesions (**D**) Early phase of green indocyanine angiography with hypocianescent choroidal lesions (**D**) Late phase of green indocyanine angiography with hypocianescent choroidal lesions and disruption of choroidal vascularization

In 2021, during the annual follow-up and despite being asymptomatic, de novo creamy multifocal choroidal lesions were detected in the posterior pole and periphery in the OD (Fig. 1A). OCT revealed significant diffuse choroidal thickening without distinct hyporreflective lesions (647 μm), along with a peripapillary neurosensory detachment (Fig. 1C). The OS showed no abnormalities (Fig. 1B and D). FA and ICG were ordered. The FA denoted areas of choroidal hypoperfusion in the early arteriovenous, venous and late phases, secondary to choroidal lesions. There was peripapillary leakage and staining consistent with neurosensory detachment, and late capillary leakage, without evidence of active vasculitis (Fig. 2A, B and C). ICG showed the presence of hypofluorescent diffuse choroidal lesions and a disorganized choroidal vascularization (Fig. 2D and E). Based on these findings, the differential diagnosis included BC reactivation versus a choroidal lymphoproliferative disorder. The unilateral nature of the lesions, the absence of inflammatory cells in the vitreous and the lack of signs of active vasculitis in the FA, were more suggestive of a choroidal lymphoproliferative process, including UHL and choroidal lymphoma (CL). Moreover, the presence of isolated subretinal fluid without cystoid macular edema is atypical in BC. An orbital and cranial magnetic resonance imaging (MRI) were ordered, with no signs of orbital or central nervous system lymphoma. The patient was referred to Hematology for evaluation, ruling out the presence of systemic lymphomatous involvement.

**Fig. 3:**
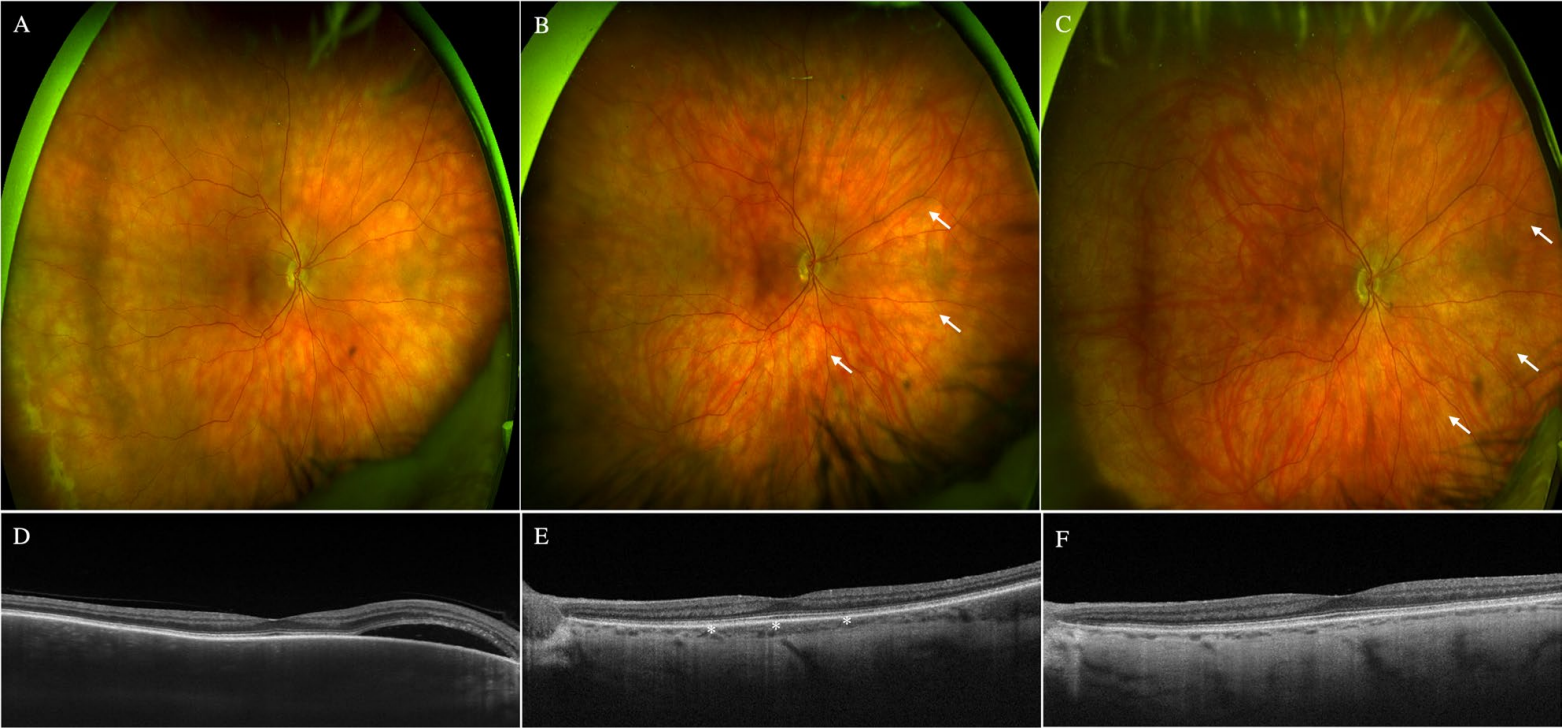
Evolution of presumed uveal lymphoid in the right eye (**A**) Retinography at presentation with multifocal creamy choroidal lesions (**B**) Retinography two weeks after completing treatment with a short regime of steroids and a better delimitation of the choroidal lesions (arrows) (**C**) Retinography at the last follow-up, 4 years after steroid treatment, with marked chorioretinal atrophy (arrows) (**D**) Macular optical coherence tomography at presentation with choroidal thickening and peripapillary serous retinal detachment (**E**) Macular optical coherence tomography two weeks after completing treatment with a short regime of steroids and choroidal thinning (asterisks) (**F**) Macular optical coherence tomography at the last follow-up, 4 years after steroid treatment, with choroidal atrophy and no signs of relapse

Hence, a choroidal biopsy was proposed to the patient, explaining the risks and benefits of the procedure to obtain a definitive diagnosis and to start a targeted treatment. However, the patient rejected the procedure, preferred close clinical follow-up and no treatment was initiated due to the preserved BVCA. 6 months later, the patient suffered from Bell’s facial palsy and was treated by Internal Medicine with oral Prednisone (initial dose of 1 mg/kg/day) and subsequent tapering for 4 weeks. Two months after completing treatment with oral prednisone, better delimitation of the choroidal lesions in the OD and an improvement in choroidal thickening up to 131 μm were observed (Fig. 3B (arrows) and 3E(asterisks)).

Eventually, ophthalmological follow-up was conducted every 6 months. After 4 years, the patient remained asymptomatic, with no alterations on OCT and cicatricial choroidal lesions in OD (Fig. 3C (arrows) and 3 F). The orbital and cranial MRI and the hematological periodical work-up did not show any abnormalities. B-mode ultrasound may also have been a reasonable imaging technique for characterizing choroidal diffuse thickening and extra-scleral involvement. Finally, although a favorable response to corticosteroid treatment may be observed in BC relapse, we suggest that the most probable finding was a presumed case of ULH in the OD in a patient with a previous history of BC.

## Discussion

This case prompted a broad differential diagnosis, encompassing infectious and inflammatory etiologies, as well as a relapse of BC, primary or secondary CL, and ULH. Several key features guided the diagnostic reasoning, including the unilateral distribution of lesions, the absence of vitreous haze, and the patient’s complete lack of symptoms. Although reactivation of BC can occur, particularly in patients not receiving chronic immunosuppression, it was deemed unlikely in this case due to the absence of bilateral involvement, vitreous or anterior chamber inflammation, and cystoid macular edema. Moreover, OCT revealed a diffuse pattern of choroidal thickening without the discrete hyporreflective lesions typically observed in BC. Importantly, the patient remained entirely asymptomatic throughout the episode. CL remained a differential consideration, particularly given the hypopigmented lesion appearance, the patient’s age, and associated choroidal thickening. Nevertheless, the combination of an asymptomatic presentation, unilateral involvement, lesion stability over five years of follow-up without scleral involvement or disease recurrence, a favorable response to a short corticosteroid regimen, and the absence of systemic symptoms or progression, supported a benign process. Consequently, the most likely diagnosis was presumed to be UHL over primary low-grade CL.

UHL is a rare condition primarily affecting middle-aged adults. It is typically unilateral and does not exhibit a predilection for either sex or race. Characterized by multifocal, static, creamy choroidal infiltrates, diffuse choroidal thickening, and pigmentary changes, it represents a polyclonal proliferation of B lymphocytes. ULH may also precede the development of CL. Hence, in a molecular and immunohistochemical study conducted by Cockerman et al., [4] 80% of enucleated eyes initially diagnosed with presumed ULH exhibited monoclonality and progression to low-grade primary CL [4]. However, this study focused on enucleated eyes with more severe clinical courses and a greater predisposition to lymphomatous transformation. Chronic infections such as Helicobacter pylori or autoimmune disorders like celiac disease, may predispose to the development of lymphoid hyperplasia and eventually to mucosa-associated lymphoid tissue (MALT) lymphoma [7, 8]. Indeed, CL exhibit histopathological similarities with ocular adnexal MALT lymphomas, including B cell lymphoid follicles with germinal centers and follicular colonization [4]. Interestingly, Ferreri et al. [9] demonstrated that up to 80% of ocular adnexal lymphomas tested positive for Chlamydia psittaci, suggesting a possible infectious etiology in the lymphoma pathogenesis. In this case, it could be hypothesized that the subclinical inflammation associated with BC could predispose to the development of ULH and eventually CL.

While evidence regarding ULH treatment remains limited, several therapeutic options are available [10]. For asymptomatic patients, close observation may constitute an appropriate management strategy, as initially adopted in our case. Enucleation is reserved for patients with severe eye pain and complete loss of vision. Corticosteroids remain the mainstay of treatment, especially for choroidal inflammation and serous retinal detachment, with initial doses ranging between 0.5 and 1 mg/kg/day followed by a gradual taper [11]. Francis et al. [12] reported the efficacy of combining corticosteroids with doxycycline to address potential associated chronic Chlamydia psittaci infections. Radiotherapy may be considered for cases with suboptimal corticosteroid response, with standard fractionated doses of 20–27 Gy14 (1.8 to 2 Gy per fraction) or ultra-low-dose (“boom-boom” radiotherapy) as utilized in CL management [13, 14].

Our patient showed a sustained response to the steroid treatment for a different extraocular pathology; however, it is important to note that the diagnosis of ULH in this case remains presumptive, as corticosteroid response can also occur in both primary CL and ULH. A definitive diagnosis of a choroidal lymphoid process would require histopathological confirmation through biopsy [3], which was not performed in this asymptomatic patient due to its refusal and to prevent potential vision loss. Instead, the presumed diagnosis was established based on findings from multimodal imaging and a comprehensive systemic evaluation.

In conclusion, ULH should be considered as a diagnostic alternative over the course of the disease in patients with BC, highlighting the need for a multidisciplinary approach to ensure accurate diagnosis, management and long-term follow-up, which are essential for monitoring disease progression and the potential transformation into CL.

## Data Availability

No datasets were generated or analyzed during the current study.
